# A CREATE-Based Educational Intervention to Improve Evidence-Based Practice Competencies in a Low-Income Country: A Feasibility Pilot Study

**DOI:** 10.3390/healthcare14172763

**Published:** 2026-09-01

**Authors:** Ilaria Marcomini, Federica Buzzi, Andrea Poliani, Federico Chiodi Daelli, Fabio Ciceri, Jorge Carlos Naranjo Alcaide, Halima Ibrahim Malik Ali Omer, Debora Rosa, Giulia Villa, Rebecca Zanuso, Mohja Marhoom, Duilio F. Manara

**Affiliations:** 1 Center for Nursing Research and Innovation, Faculty of Medicine and Surgery, Vita-Salute University, 20132 Milan, Italy; marcomini.ilaria@unisr.it (I.M.); rosa.debora@unisr.it (D.R.); villa.giulia@unisr.it (G.V.); cenri@unisr.it (D.F.M.); 2 IRCCS San Raffaele Scientific Institute, 20132 Milan, Italy; buzzi.federica@hsr.it (F.B.); ciceri.fabio@hsr.it (F.C.); 3 Italian Association for Solidarity Among Peoples (AISPO), 20132 Milan, Italy; chiodidaelli.federico@hsr.it (F.C.D.); zanuso.rebecca@hsr.it (R.Z.); 4 Comboni College of Science and Technology, Port Sudan 33311, Sudan; naranjoalcaide@hotmail.com (J.C.N.A.); hsmith.ali@gmail.com (H.I.M.A.O.); mohja.3@gmail.com (M.M.)

**Keywords:** evidence-based practice, low-income countries, maternal and child health, nursing education, remote education, Sudan

## Abstract

**Highlights:**

**What are the main findings?**

- A 40 h synchronous online educational program based on the CREATE framework was feasible for delivering evidence-based practice training to nursing students and registered nurses in Sudan.
- The intervention was associated with significant improvements in overall evidence-based practice competencies in both groups, with gains in knowledge among students and nurses, attitudes among students, and skills among registered nurses.

**What are the implications of the main findings?**

- Synchronous distance learning, when supported by local institutional collaboration and cultural mediation, may represent a practical strategy to strengthen evidence-based practice capacity in resource-constrained and conflict-affected settings.
- Linking evidence-based practice education to a locally relevant clinical activity, such as the development of a Kangaroo Mother Care protocol, may enhance participants’ engagement and support the translation of evidence into practice.

**Abstract:**

Background: Evidence-based practice (EBP) competencies remain limited in many low-income countries, where access to research training is often constrained by limited educational resources and humanitarian crises. This study evaluated the feasibility and preliminary effectiveness of a CREATE-based teaching strategy to improve EBP competencies among nurses and nursing students in Sudan. Methods: A quasi-experimental pre–post study without a control group was conducted among 29 participants (17 registered nurses and 12 nursing students) at Comboni College, Port Sudan. The intervention consisted of a 40 h synchronous online educational program based on the CREATE framework, integrating interactive lectures, practical group exercises, teach-back activities, and the collaborative development of an evidence-based Kangaroo Mother Care protocol. EBP competencies were assessed before and after the intervention using the EBP-COQ and EBP-COQ Prof© questionnaires. Paired-sample *t*-tests were used to compare pre- and post-intervention scores. Results: Nursing students demonstrated significant improvements in knowledge (*p* = 0.009), attitudes (*p* = 0.015), and overall EBP competency (*p* = 0.006), whereas changes in self-reported skills were not significant. Registered nurses showed significant improvements in knowledge (*p* < 0.001), skills (*p* < 0.001), and overall EBP competency (*p* < 0.001), while changes in attitudes and EBP utilization were not statistically significant. Conclusions: This pilot study suggests that a CREATE-based teaching strategy delivered through synchronous distance learning is feasible and may improve EBP competencies among nurses and nursing students in Sudan, despite the challenges of a resource-constrained and conflict-affected setting. Larger controlled studies are needed to confirm these findings and evaluate long-term educational and clinical outcomes.

## 1. Introduction

Evidence-based practice (EBP) is a cornerstone of safe, effective, and high-quality healthcare, supporting clinical decision-making through the integration of the best available evidence, professional expertise, patient preferences, values, and contextual factors [1,2]. In nursing, EBP contributes to reducing unwarranted practice variation, improving care quality, strengthening professional accountability, and translating research findings into clinical practice [3].

Despite its importance, EBP implementation remains challenging, especially in low- and middle-income countries (LMICs). Nurses in these settings often face limited access to information, inadequate staffing, lack of institutional support, insufficient time, poor integration between academic and clinical environments, and limited EBP knowledge and skills [3]. Although attitudes toward EBP are frequently positive, familiarity, knowledge, skills, and actual implementation are often moderate, low, or suboptimal [4]. These barriers indicate that EBP implementation requires not only individual competence but also organizational support, accessible resources, and context-sensitive educational strategies.

Teaching EBP also requires approaches that move beyond theoretical instruction. Effective educational programs should support learners in applying the full EBP process, including asking answerable questions, searching for evidence, appraising evidence, integrating findings into practice, and evaluating outcomes. Educational interventions combining lectures, group discussions, practical exercises, and hands-on activities have been shown to improve nurses’ EBP knowledge, skills, attitudes, confidence, and behaviors [5]. In LMICs, continuing nursing education is particularly important when it incorporates multiple teaching modalities, strong local partnerships, nurse empowerment, repeated learning opportunities, and cultural adaptation [6].

Previous research indicates that EBP education can also be successfully implemented in resource-constrained settings. Educational interventions conducted in LMICs have reported improvements in nurses’ EBP knowledge, skills, attitudes, and competencies, including through web-based, competency-based, and multimodal training approaches [6,7]. However, available interventions remain heterogeneous in their content, delivery methods, assessment strategies, and targeted EBP outcomes, and evidence regarding their implementation in fragile or humanitarian health systems remains limited.

One approach to structuring and evaluating EBP education is the Classification Rubric for EBP Assessment Tools in Education (CREATE) framework, which organizes EBP learning around five sequential components: ask, search, appraise, integrate, and evaluate; and distinguishes educational outcomes such as attitudes, knowledge, skills, and behavior [8]. CREATE was originally developed primarily as a taxonomy for classifying and aligning EBP assessment tools rather than as a prescriptive educational intervention. Nevertheless, its structure provides a useful framework for aligning educational content, learning activities, and assessment with the complete EBP process. To our knowledge, evidence describing structured CREATE-aligned educational programs for nursing students and practicing nurses in LMICs remains limited, particularly when education is delivered remotely in settings affected by armed conflict and severe health-system disruption. This represents an important gap because the feasibility and educational effects observed in comparatively stable settings cannot necessarily be assumed to translate directly to humanitarian contexts.

In Sudan, the need for targeted EBP education is reinforced by a fragile and overburdened health system. Since the escalation of armed conflict in April 2023, displacement, disruption of health services, and increased humanitarian needs have further strained local healthcare capacity [9]. Port Sudan, in Red Sea State, has assumed a central administrative and humanitarian role, increasing pressure on public services [10]. Red Sea State is characterized by dispersed settlements, rural and nomadic populations, and geographical barriers that may hinder access to basic services, making health workforce strengthening especially relevant [11]. Available evidence also suggests important EBP-related gaps among Sudanese nurses, with poor knowledge, skills, and attitudes, and limited previous EBP training reported in hospital settings [8]. Against this background, Sudan represents not merely a geographical setting in which to replicate an existing educational approach, but a context in which the feasibility of delivering structured EBP education under conditions of conflict, displacement, constrained resources, and limited access to conventional educational infrastructure requires specific evaluation. This is particularly relevant in maternal and newborn care, where the successful implementation of evidence-based interventions can contribute significantly to improving outcomes even in resource-constrained environments [12]. Within maternal and child healthcare, kangaroo mother care (KMC) represents a relevant example of an evidence-based intervention suitable for resource-constrained settings. KMC is recommended as routine care for preterm or low-birth-weight infants, highlighting its role in reducing mortality and hypothermia [13]. To address these educational needs and support the implementation of evidence-based maternal and child healthcare practices, a targeted educational initiative was developed within an international cooperation project led by AISPO (Italian Association for Solidarity among Peoples). The educational program, part of the entire mission, aimed to strengthen nursing students’ and registered nurses’ competencies in research methodology and EBP with specific attention to maternal and child healthcare. The final applied activity, focused on developing an internal protocol on KMC, was designed to connect EBP learning with a locally relevant clinical intervention and support evidence translation into practice.

Accordingly, the present pilot study aimed to evaluate the feasibility and preliminary effectiveness of a synchronous distance educational program, structured according to the CREATE framework, in improving EBP-related competencies among Bachelor of Science in Nursing students and registered nurses in Red Sea State, Sudan, using a single-arm pre–post design.

## 2. Methods

### 2.1. Study Design

A quasi-experimental pre–post study without a control group was conducted to evaluate the effect of a structured educational program on participants’ knowledge, attitudes, and reported use of evidence-based practice. The study was developed as part of the AISPO (Italian Association for Solidarity among Peoples) international cooperation project “Support to Public Health Services for Women and Children in the Red Sea, Kassala and Khartoum States”—SESAMAI, AID 12513—which aimed to strengthen maternal and child healthcare services in Sudan. Because of the ongoing security situation in Sudan and the impossibility for the Italian teaching team to travel to the country safely, the educational program was specifically designed and delivered through a synchronous online learning format. This approach ensured continuity of training activities despite travel restrictions and security-related constraints while maintaining direct interaction between participants and instructors.

**Figure 1:**
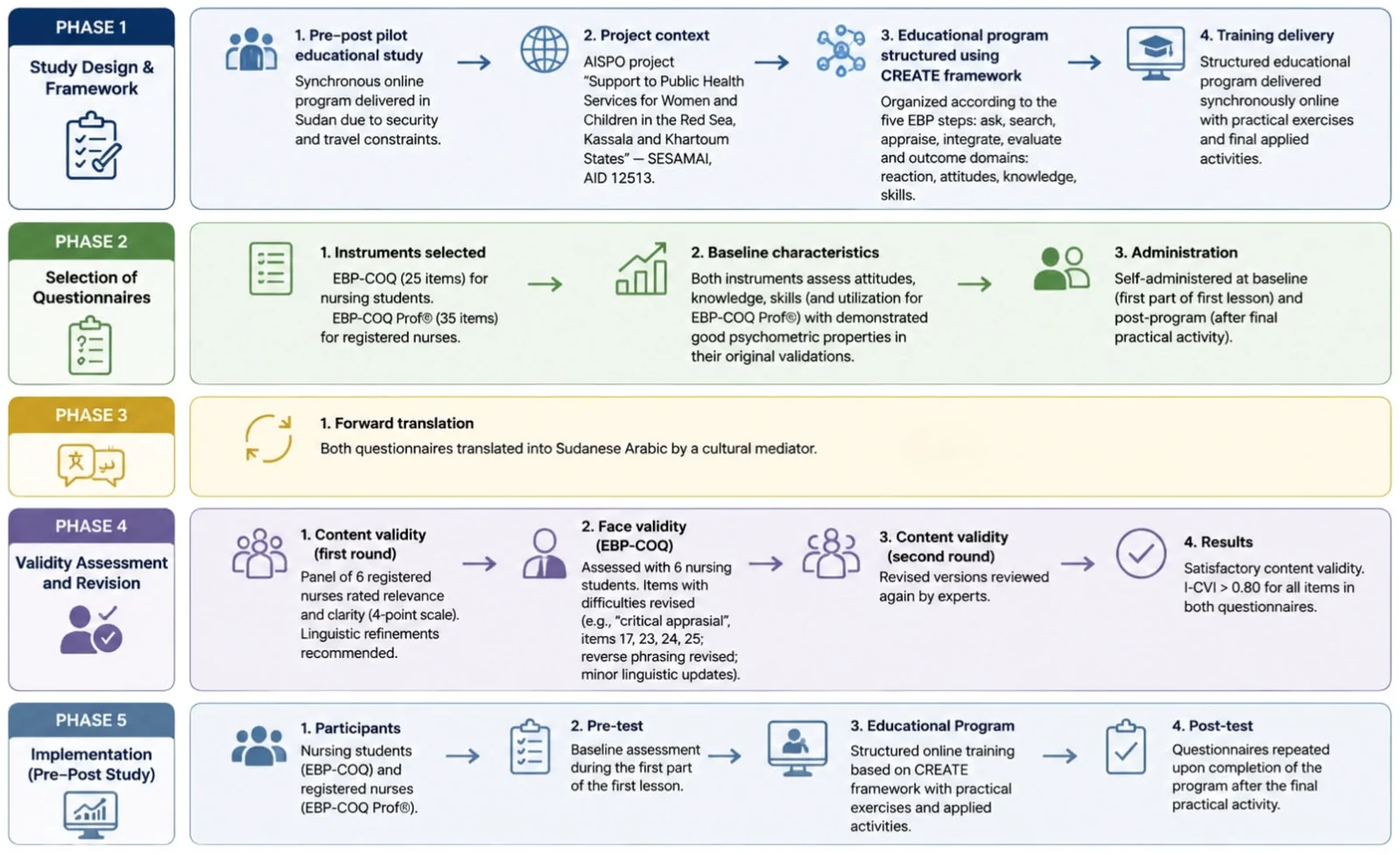
Flow diagram of the entire program process.

The educational program was designed to be consistent with the Classification Rubric for EBP Assessment Tools in Education (CREATE) framework [14]. The CREATE framework provides a taxonomy for classifying EBP educational assessment tools according to the five steps of EBP: ask, search, appraise, integrate, and evaluate, and according to different educational outcome domains, including reaction to the educational experience, attitudes, knowledge, and skills. In this study, CREATE was used not only as a reference framework for assessment but also as a pedagogical structure to organize the training contents, practical exercises, and final applied activities. The entire educational program is shown in detail in Figure 1.

### 2.2. Questionnaires Used for the Pre–Post Study

The pre–post assessment was conducted using two self-administered evidence-based practice (EBP) questionnaires. Nursing students completed the Evidence-Based Practice Competence Questionnaire (EBP-COQ) [15], while registered nurses completed the Evidence-Based Practice Competency Questionnaire for Professionals (EBP-COQ Prof©) [16]. Both instruments were administered at baseline during the first part of the first lesson and repeated upon completion of the educational program, following the final practical activity. Prior to their use, formal authorization was obtained from the original authors, and a use agreement was signed. The EBP-COQ is a 25-item questionnaire developed to assess undergraduate nursing students’ self-perceived EBP competence across three dimensions: attitudes, skills, and knowledge. In its original validation, the instrument showed good internal consistency, with a Cronbach’s alpha of 0.888 for the total scale and values of 0.940, 0.756, and 0.800 for the three subscales, respectively. The three-factor structure explained 55.55% of the variance.

The EBP-COQ Prof© is a 35-item questionnaire designed for registered nurses and evaluates four dimensions of EBP competency: attitudes, knowledge, skills, and utilization. In the original validation study, the instrument demonstrated good psychometric properties, with Cronbach’s alpha values ranging from 0.817 to 0.948 across dimensions and adequate construct validity supported by exploratory and confirmatory factor analyses.

Both questionnaires were translated into Sudanese Arabic through a forward-translation process conducted by a cultural mediator. The translated versions were then independently back-translated into the original language and compared with the original questionnaires to verify semantic and conceptual equivalence and ensure that the meaning of the items had been preserved. Any discrepancies identified during this process were discussed and resolved before proceeding to the validation phase.

The translated versions subsequently underwent a first-round content and face-validity assessment.

Content validity was evaluated by a panel of six registered nurses who independently rated the relevance and clarity of each item using a 4-point scale. Overall, reviewers considered both the EBP-COQ and EBP-COQ Prof© appropriate for the target population. However, several linguistic refinements were recommended to improve clarity and conceptual equivalence with the original instruments. In particular, some EBP-related technical terms were replaced with more commonly used expressions in Sudanese Arabic, ambiguous wording was simplified, and selected items were rephrased to better convey the intended meaning. Reviewers also highlighted that respondents with limited previous exposure to EBP might experience difficulties understanding questions related to EBP concepts and research terminology.

Face validity was assessed separately among six nursing students using the EBP-COQ. Although the questionnaire was generally considered understandable, students reported difficulty interpreting several items. Based on their feedback, the translation of “critical appraisal” was revised from a literal translation to a more explanatory expression reflecting the process of systematically evaluating research evidence. Additional modifications included clarifying items 17, 23, 24, and 25 through simpler wording and sentence restructuring. Furthermore, the reverse-worded item “I prefer to avoid reading scientific articles” was rephrased to reduce potential misunderstanding associated with negative sentence construction. Minor spelling corrections and linguistic adjustments were also implemented throughout both questionnaires to improve readability and consistency.

Following these revisions, both questionnaires underwent a second round of expert review. As part of this final review, the revised Sudanese Arabic versions were again checked against the original instruments to confirm that the linguistic adaptations had not altered the intended meaning of the items. The revised versions demonstrated satisfactory content validity, with item-level Content Validity Index (I-CVI) values exceeding the recommended threshold of 0.80 for all items in both the EBP-COQ and EBP-COQ Prof©. The final Sudanese Arabic versions were therefore considered linguistically clear, culturally appropriate, and conceptually equivalent to the original instruments and were subsequently administered to participants enrolled in the educational program. Internal consistency was assessed using Cronbach’s alpha coefficient on baseline (pre-intervention) data. The EBP-COQ Professional Competence Scale demonstrated excellent reliability, with an overall Cronbach’s alpha of 0.912, indicating a high degree of internal consistency among the items.

The Evidence-Based Practice Questionnaire for Nursing Students showed moderate overall internal consistency, with a Cronbach’s alpha of 0.663. Although lower than that observed for the EBP-COQ Professional Competence Scale, this value suggests a moderate level of reliability within the study sample.

In addition to the questionnaire items, participants were asked to provide socio-demographic and professional information. For nursing students, data included age, gender, year of study, and previous participation in EBP-related training. For registered nurses, information collected included age, gender, educational level, years of professional experience, clinical area of practice, and previous EBP training.

### 2.3. Setting

The study was conducted at Comboni College in Port Sudan, located in north-eastern Sudan, in the Red Sea State. Comboni College of Science and Technology is a higher education institution originally located in Khartoum, Sudan, that was forced to relocate to Port Sudan due to the ongoing armed conflict. It offers several academic programs, including a Bachelor of Science in Nursing, and served as the local partner for the educational and research program. For the intervention, a dedicated meeting room equipped with tables and chairs suitable for lectures and group-based activities was made available. The room had internet access, a slide-projection screen and screen-sharing capability, audio speakers, and a meeting microphone that enabled interaction between participants and remote teachers. A movable camera was positioned on the screen and oriented toward the meeting room, enabling remote teachers to observe participants’ activities and interact with them during the sessions. The room was ventilated and equipped with air conditioning and fans to ensure a comfortable learning environment during the sessions, particularly considering the high local temperatures and the number of participants attending the lessons in the same room.

Ten internet-enabled laptop computers were available for the training activities. Based on this availability, the maximum number of participants was set at 30, with an average ratio of three participants per laptop.

### 2.4. Participants and Sampling

A convenience sample of 29 participants was recruited. The sample included registered nurses and Bachelor's Degree in Nursing students. The students were enrolled in the Bachelor’s Degree in Nursing program at Comboni College, while the registered nurses came from various hospitals and community healthcare services in the Red Sea State area and were mainly engaged in maternal and child health, consistent with the international cooperation project’s focus on these two health fields. The educational initiative was disseminated through Comboni College, and participation was based on an expression of interest. The final sample consisted of the 29 nursing students and registered nurses who responded to the call and volunteered to participate in the educational program. The inclusion of both nursing students and professionals was based on the educational needs identified in the local context. In particular, research methodology and EBP are not systematically or extensively included in nursing education and professional training pathways. Therefore, the program was designed to provide both students and registered nurses with foundational and applied competencies in nursing research methodology and evidence-based practice.

### 2.5. Educational Framework and Program Structure

The educational program was structured according to the CREATE framework [14]. This framework was used to align the learning objectives, teaching strategies, practical exercises, and assessment methods with the main dimensions of EBP learning. In line with CREATE, the program addressed the five core steps of EBP: asking answerable clinical or research questions, searching for the best available evidence, appraising evidence, integrating evidence into practice, and evaluating its application.

Participants were introduced to the complete EBP process, including asking answerable clinical or research questions, searching for the best available evidence, critically appraising evidence, integrating evidence into practice, and evaluating the outcomes of evidence implementation. However, the structured practical activities conducted during the program primarily focused on the Ask, Search, Appraise, and Integrate steps. The Evaluate step was addressed conceptually but could not be directly practiced, as this would have required implementation of the developed protocol in clinical practice and subsequent assessment of its outcomes.

The program also targeted several CREATE educational outcome domains. Knowledge was addressed through theoretical lessons on research methodology, EBP principles, study designs, data collection, and evidence appraisal. Skills were developed through practical exercises, including literature searches, database construction, basic statistical analysis, qualitative data analysis, and evidence synthesis. Attitudes and self-efficacy were addressed through guided discussion, reflection, and teach-back activities to strengthen participants’ confidence in using EBP in clinical contexts. Behavioral change was promoted through the final applied activity, in which participants developed an internal protocol on kangaroo mother care to support the translation of evidence into local clinical practice.

This structure is consistent with the CREATE framework, which distinguishes among different levels of EBP learning, from learners’ reactions and attitudes to knowledge, skills, behaviors, and potential patient benefits. The framework also emphasizes the importance of linking assessment methods to learner characteristics, learning aims, and the specific EBP steps addressed by the educational intervention.

### 2.6. Educational Intervention

Participants attended a synchronous distance educational program delivered through Microsoft Teams (version 26120.3106.4725.800). The program consisted of 40 h of training. Teaching hours were scheduled according to Central European Time (CET). Due to the time difference, sessions initially corresponded to 10:00 a.m.–2:00 p.m. in Sudan; after the standard time change in Italy, the sessions corresponded to 9:00 a.m.–1:00 p.m. in both Italy and Sudan. Most sessions lasted 4 h. The only exception was the final training module, which consisted of 16 h delivered over three consecutive days: one full 8 h day and two morning sessions from 9:00 a.m. to 1:00 p.m. Each session included an intermediate break to allow participants to rest from the lesson, recover energy by eating, and have time for prayer, in accordance with participants’ needs and the local cultural and religious context.

The training took place weekly between March and May 2026. The weekly schedule was intentionally adopted to allow participants time to reflect on, internalize, and consolidate the contents addressed during each session before attending the next one. Access to the lessons was provided through a Microsoft Teams link sent by email to Comboni College.

All teaching materials, including slides, exercises, and supporting documents, were uploaded to a shared Google Drive folder before each lesson to facilitate participants’ access. The folder also included the reference bibliography for the course. Access to the shared folder was restricted to participants and educational staff, including teachers, facilitators, and the cultural mediator.

### 2.7. Teaching Team

The educational program was delivered by a team of Italian nursing researchers. Two nurse research fellows alternated in teaching activities and ensured continuity throughout the course. One teacher was a female nurse researcher with a PhD, while the other was a male nurse researcher and PhD student.

In addition, a female nurse with expertise in pediatric, maternal, and child healthcare and holding a Master’s Degree in Nursing and Midwifery Sciences was invited to participate in the educational program. Her contribution was particularly relevant for providing practical examples and clinical applications of the theoretical content. This allowed the program to maintain a close connection between research methodology, EBP, and the broader objectives of the international cooperation project in maternal and child healthcare.

### 2.8. Language, Translation, and Cultural Mediation

All lessons were delivered in English and translated into Sudanese Arabic by a cultural mediator with a Bachelor’s degree in English Language and Literature. The translation process was further supported by a local physician, who assisted and facilitated the mediator in clarifying specific medical, nursing, and healthcare-related terminology. This approach was adopted to facilitate participants’ understanding and to ensure that technical concepts were accurately conveyed within the local linguistic and cultural context.

### 2.9. Educational Intervention Contents

**Figure 2:**
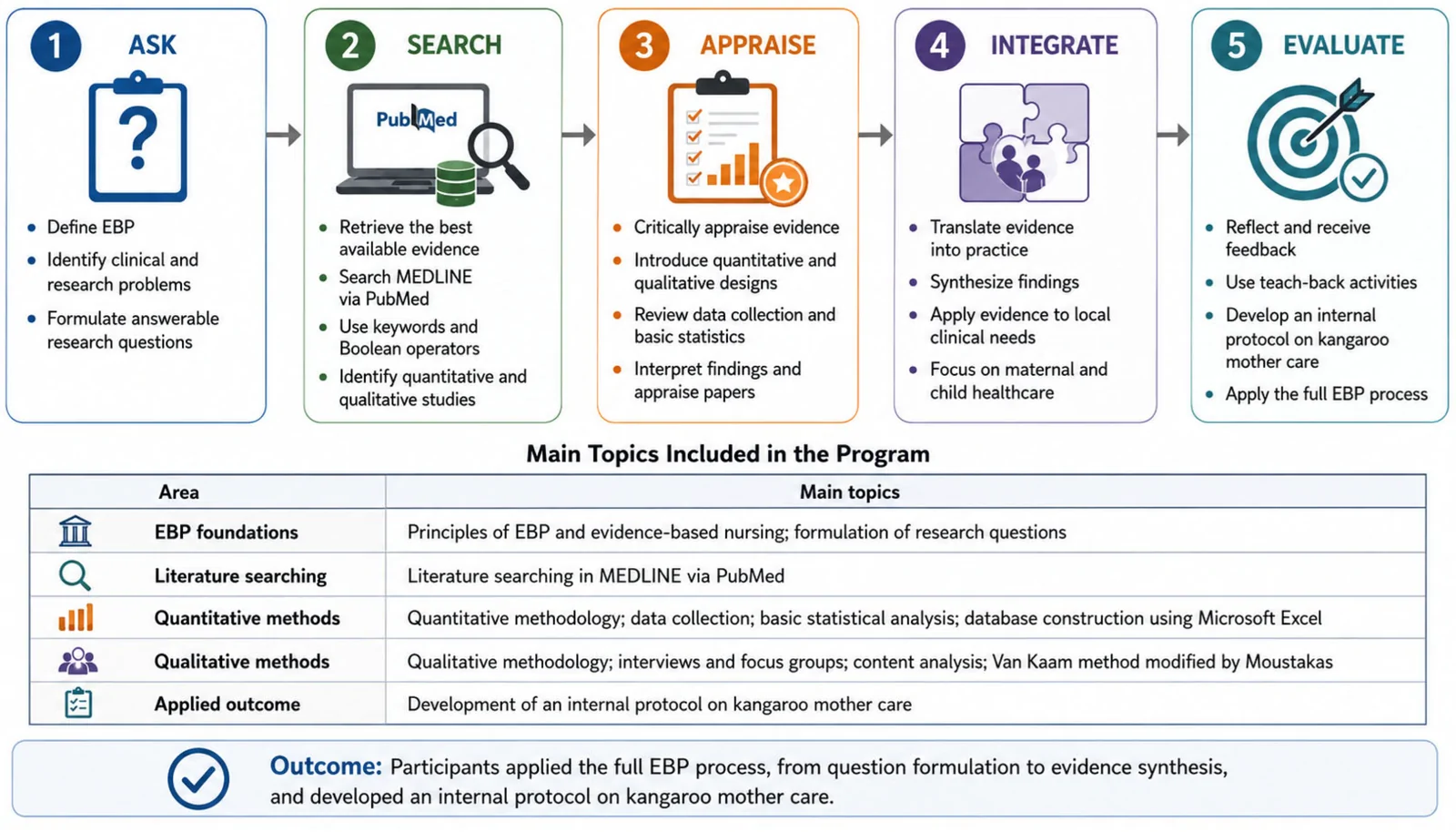
Graphical synthesis of the educational program using the CREATE framework.

The contents of the educational program were organized according to the five EBP steps described in the CREATE framework (Figure 2).

First, the “Ask” component focused on the definition of EBP, the identification of clinical and research problems, and the formulation of answerable research questions. Participants were guided in translating practical problems into structured questions suitable for evidence searching.

Second, the “Search” component focused on identifying and retrieving the best available evidence. Participants were trained to search MEDLINE via PubMed, to use keywords and Boolean operators, and to identify relevant quantitative and qualitative studies.

Third, the “Appraise” component addressed the critical appraisal of evidence. Participants were introduced to quantitative and qualitative research designs, data collection methods, basic statistical concepts, and the interpretation of research findings. They also worked on the appraisal of papers retrieved during practical exercises.

Fourth, the “Integrate” component focused on the translation of evidence into practice. Participants were guided in synthesizing evidence and considering how research findings could be applied to local clinical needs, with particular attention to maternal and child healthcare.

Finally, the “Evaluate” component, defined as the assessment of outcomes following the implementation of evidence in clinical practice, was introduced conceptually during the educational program but was not directly practiced. The final applied activity concluded with the development of an evidence-based internal protocol on kangaroo mother care and therefore involved the Ask, Search, Appraise, and Integrate steps. Implementation of the protocol and subsequent evaluation of clinical or patient-level outcomes were beyond the scope and timeframe of the present educational intervention. Reflection, feedback, and teach-back were used throughout the program as educational strategies to reinforce learning and assess participants’ understanding, rather than as activities representing the Evaluate step of EBP. The main topics included in the program are summarized in Table 1 and include: principles of EBP and evidence-based nursing; formulation of research questions; literature searching in MEDLINE via PubMed; quantitative methodology; data collection; basic statistical analysis and database construction using Microsoft Excel; qualitative methodology; interviews and focus groups; content analysis; and the Van Kaam method modified by Moustakas.

### 2.10. Practical Exercises and Group Work

The program combined theoretical lessons with practical exercises. After the theoretical component of each lesson, participants engaged in group-based activities lasting approximately 20–30 min each. These exercises were designed to consolidate the contents covered during the session and to support the acquisition of practical EBP skills.

Participants were divided into three groups of approximately 10 members each. Groups were balanced in terms of number of participants and composition, including both registered nurses and nursing students, gender, and expertise in maternal and pediatric areas. When computer-based activities were required, such as literature searching, database construction, basic statistical analysis, or qualitative data analysis, each group had access to three computers. This organization allowed all participants to practice the use of computers and digital tools during the exercises.

Feedback was provided through the evaluation of practical activities and through the teach-back method. During the lessons, teachers used guiding questions to recall previously addressed concepts, verify understanding, and stimulate reflection on the EBP process. These strategies were intended to reinforce knowledge, support self-efficacy, and promote the gradual development of EBP-related skills.

### 2.11. Final Activity

The educational program concluded with three practical meetings focused on the development of an internal protocol on kangaroo mother care. These meetings were mainly guided by the nurse with expertise in maternal and child healthcare.

During this final activity, participants worked in groups to develop a protocol that could be applied and implemented within their own clinical units. The protocol focused on the use, benefits, indications, and precautions related to kangaroo mother care. To develop the protocol, participants formulated a research question, searched the literature in MEDLINE via PubMed, critically appraised the retrieved papers, and synthesized the available evidence to answer the research question.

Both quantitative and qualitative studies emerged during the literature search. These studies were used not only to support protocol development but also to reinforce previously addressed course contents. Through teach-back activities, participants were encouraged to revisit and apply key concepts related to EBP, research methodology, critical appraisal, and evidence synthesis.

### 2.12. Access to Materials

All synchronous lessons were video-recorded. Recordings included the teacher facilitation from Milan, the classroom in Port Sudan, and the slides presented during each session. After each lesson, the video recording was uploaded to the shared Google Drive folder, allowing participants to review the contents and listen to the lessons again after the synchronous session.

### 2.13. Ethical Aspects

This pre–post pilot study was conducted in accordance with the principles of the Declaration of Helsinki. Before implementation, the training activities were formally communicated to and approved by the competent local health authority. In the study setting, the Ministry of Health and Social Development also acts as the ethical approving authority. Accordingly, the Director of the Health Sector Office of the Ministry of Health and Social Development, Red Sea State, Sudan, issued an official no-objection and approval letter for the educational program, reference no. 44/B/1, dated 23 February 2026. Participation in the educational activities and related evaluation procedures was voluntary. Participants were informed about the purpose of the training and the use of aggregated data for scientific and educational purposes. No clinical interventions were performed, and no biological samples or identifiable clinical records were collected. Data were analyzed and reported anonymously and in aggregate form to ensure participants’ confidentiality.

### 2.14. Statistical Analysis

Data were analyzed using IBM SPSS Statistics version 29 (IBM Corp., Armonk, NY, USA). Descriptive statistics were used to summarize participants’ characteristics separately for nursing students and registered nurses, including socio-demographic characteristics for both groups, clinical training characteristics for students, and professional and clinical practice characteristics for nurses. Continuous variables were reported as means and standard deviations (SD), whereas categorical variables were presented as frequencies and percentages.

For both the EBP-COQ and EBP-COQ Prof©, total and domain scores were calculated according to the scoring procedures described by the original authors.

Prior to inferential analyses, the normality of pre–post difference scores was evaluated using the Shapiro–Wilk test. To evaluate changes in EBP-related competencies following the educational intervention, pre- and post-intervention scores were compared using paired-samples *t*-tests. Separate analyses were conducted for nursing students (EBP-COQ) and registered nurses (EBP-COQ Prof©). Differences were examined for both total questionnaire scores and individual domain scores.

For statistically significant comparisons, effect sizes were calculated using Cohen’s d for paired samples and interpreted according to conventional thresholds as small (0.20), medium (0.50), and large (0.80). Mean differences and corresponding 95% confidence intervals (95% CI) were also calculated to quantify the magnitude and precision of the observed changes.

All statistical tests were two-tailed, and a *p*-value < 0.05 was considered statistically significant.

## 3. Results

### 3.1. Characteristics of Participants

The socio-demographic and professional characteristics of the participants are presented in Table 1. A total of 29 participants were included in the study, comprising 12 nursing students and 17 registered nurses. Nursing students had a mean age of 24.2 years (SD = 4.1), and most were female (66.7%). All students were enrolled in a Bachelor of Nursing program, and two-thirds (66.7%) reported having observed the implementation of EBP during their clinical placements. Most students resided in Port Sudan (66.7%).

Registered nurses had a mean age of 37.6 years (SD = 10.2) and an average of 14.4 years of professional experience (SD = 10.0). The majority were female (88.2%) and held a Bachelor’s degree in Nursing (76.5%), while 23.5% had completed postgraduate education (Master’s degree or PhD). Most nurses resided in Port Sudan (76.5%). Regarding clinical practice settings, 41.2% worked in critical care units and 47.1% were employed in maternal and child health services.

A total of 12 nursing students completed both the pre- and post-intervention assessments. Assumptions for paired-sample analyses were met, as the distribution of difference scores did not significantly deviate from normality (Shapiro–Wilk *p* > 0.05).

As shown in Table 2, for the Attitude dimension, mean scores increased from 51.67 ± 4.38 at baseline to 55.25 ± 3.08 after the intervention. The mean increase was 3.58 points (95% CI 0.85–6.31), representing a statistically significant improvement (paired *t*(11) = −2.89, *p* = 0.015).

For the Skills dimension, scores changed from 17.33 ± 3.55 at baseline to 16.92 ± 2.57 post-intervention. The mean difference was −0.42 points (95% CI −2.18 to 1.35), indicating no statistically significant change (paired *t*(11) = 0.52, *p* = 0.614). For the Knowledge dimension, mean scores increased from 15.33 ± 4.77 to 20.08 ± 2.02 following the intervention. The mean improvement was 4.75 points (95% CI 1.43–8.07), which was statistically significant (paired *t*(11) = −3.15, *p* = 0.009). Regarding the Overall Total Score, participants showed a significant increase from 84.33 ± 7.73 at baseline to 92.25 ± 3.72 after the intervention. The mean increase was 7.92 points (95% CI 2.79–13.05), corresponding to a statistically significant improvement (paired *t*(11) = −3.40, *p* = 0.006). Table 3 shows the pre-post comparison of outcome measures.

### 3.2. Nurses' Scores

A total of 17 registered nurses completed both the pre- and post-intervention assessments (Table 4). The distribution of the pre–post difference scores was examined using the Shapiro–Wilk test, and no significant deviation from normality was detected (*p* > 0.05). For the Attitude domain, mean scores increased from 36.35 ± 2.89 at baseline to 37.53 ± 1.84 following the intervention. The mean increase was 1.18 points (95% CI: −0.47 to 2.82). However, this change did not reach statistical significance (paired *t*(16) = −1.51, *p* = 0.150). For the Knowledge domain, scores increased substantially from 18.53 ± 5.44 to 29.88 ± 3.20. The mean improvement was 11.35 points (95% CI: 8.68 to 14.02), representing a statistically significant increase (paired *t*(16) = −9.01, *p* < 0.001). Similarly, the Skills domain showed a significant increase from 27.18 ± 5.73 at baseline to 35.76 ± 3.99 post-intervention. The mean difference was 8.59 points (95% CI: 5.61 to 11.56), corresponding to a statistically significant improvement (paired *t*(16) = −6.12, *p* < 0.001). For the Utilization domain, mean scores increased from 42.18 ± 9.13 to 46.06 ± 8.45 after the intervention. The mean difference was 3.88 points (95% CI: −3.26 to 11.02). This change was not statistically significant (paired *t*(16) = −1.15, *p* = 0.266).

Finally, the Overall Total Score increased significantly from 124.24 ± 17.32 at baseline to 149.24 ± 13.78 following the intervention. The mean improvement was 25.00 points (95% CI: 14.20 to 35.80), corresponding to a statistically significant difference (paired *t*(16) = −4.91, *p* < 0.001).

## 4. Discussion

This study evaluated the preliminary effect of a structured educational intervention based on the CREATE framework [14] in improving EBP competencies among nursing students and registered nurses in a low-income country affected by humanitarian and health-system challenges. There are some studies in Sudan concerning the knowledge, attitude and practice of evidence-based medicine education [8,17], but to our knowledge, there is no study concerning EBP educational interventions. Therefore, the present experience may provide a useful model for the country and for other LMICs, particularly those affected by conflict, instability, and limited educational resources.

Our results showed that both registered nurses and nursing students experienced statistically significant improvements in overall EBP competency scores following the intervention. These findings indicate that the CREATE-based educational program was effective in strengthening EBP competencies across participants with different levels of professional experience. Furthermore, they support the potential applicability of this educational model in resource-constrained and fragile healthcare settings, where opportunities for formal EBP training are often limited.

The findings also highlight the value of contextualized and practice-oriented educational approaches. Rather than focusing solely on theoretical EBP concepts, the program incorporated a hands-on activity involving the development of a KMC protocol, thereby linking EBP learning to a locally relevant maternal and newborn health priority [13]. This approach may have facilitated participants’ engagement with the EBP process and strengthened the perceived relevance of evidence-informed decision-making within their clinical context.

A particularly important finding was the significant improvement in EBP knowledge observed among both nursing students and registered nurses. Baseline scores confirmed the existence of important knowledge gaps, consistent with previous evidence from Sudan reporting limited EBP preparation among nurses and inadequate exposure to research methodology and evidence-based practice during training [8]. Following the intervention, knowledge scores increased significantly in both groups, suggesting that the educational program was successful in strengthening participants’ understanding of EBP concepts and processes. These findings reinforce the need to strengthen the integration of research methodology and EBP content within undergraduate nursing curricula and continuing professional development programs [3,4].

The different pattern of results observed between students and professionals is also noteworthy. Nursing students demonstrated significant improvements in knowledge, attitudes toward EBP, and overall EBP competence, whereas registered nurses showed significant gains in knowledge, skills, and overall competence. The significant increase in attitudes among students may reflect the influence of educational exposure during a formative stage of professional development, when beliefs and perceptions regarding evidence-based care may be more readily shaped. Previous studies have shown that EBP-related attitudes and beliefs among nursing students are influenced by curricular exposure and educational experiences, supporting the importance of introducing EBP competencies early in professional training [18]. In contrast, attitudes among registered nurses were already relatively positive at baseline, leaving less room for measurable improvement. The absence of a statistically significant change in this domain may therefore reflect a ceiling effect rather than a lack of educational impact.

The significant improvement in EBP skills observed among registered nurses deserves particular attention. Experienced clinicians may be better positioned to immediately connect newly acquired EBP concepts with real clinical situations and decision-making processes encountered in practice. Professional experience likely facilitates the translation of theoretical knowledge into practical competencies such as literature searching, critical appraisal, and evidence application. Among nursing students, EBP skills remained substantially unchanged, with only a minimal and non-statistically significant decrease observed after the intervention. This small reduction may partly reflect a more critical self-assessment of EBP skills following the educational program. As students gained greater knowledge of EBP concepts and processes, they may have developed a clearer understanding of the competencies required to apply EBP in practice and consequently evaluated their own skills more cautiously. However, given the very small magnitude of the change and the wide confidence interval including zero, this interpretation remains tentative, and the finding should not be considered evidence of an actual deterioration in EBP skills. In addition, practical EBP competencies may require longer-term exposure, repeated practice, and opportunities for application in clinical or simulated settings before measurable improvements become evident [18].

Another important finding concerns the utilization dimension among registered nurses. Although utilization scores increased following the intervention, the improvement did not reach statistical significance. This result is consistent with the broader literature suggesting that changes in EBP implementation are more difficult to achieve than changes in knowledge, attitudes, or skills [3]. Educational interventions can enhance individual competence; however, the translation of competence into routine clinical practice often depends on organizational and contextual factors, including leadership support, workload, access to scientific resources, mentorship opportunities, and institutional culture.

### Limitations

This study has several limitations that should be considered when interpreting the findings. First, the pre–post design without a control group limits the ability to establish causal relationships between the intervention and the observed improvements in EBP competencies. Therefore, the observed changes cannot be attributed exclusively to the intervention, as testing effects, increased familiarity with the questionnaires, participant motivation, or Hawthorne effects may also have contributed to the improvements. Second, the relatively small sample size may limit the generalizability of the findings. In addition, the student questionnaire showed moderate internal consistency (Cronbach’s α = 0.663), below the commonly accepted threshold of 0.70. This may have affected measurement reliability and should be considered when interpreting the observed changes among nursing students. Third, although the educational activities were conducted in English, communication and interaction were frequently facilitated by a cultural mediator who provided linguistic and contextual support. While this approach was essential to ensure participants’ engagement and comprehension, it may have influenced the delivery and interpretation of some educational content. Finally, all outcomes were assessed using self-report questionnaires and therefore reflect participants’ perceived EBP competencies rather than objectively demonstrated performance. This may have introduced self-report bias and limits conclusions regarding actual changes in EBP competencies. Future studies should complement self-reported measures with objective or performance-based assessments.

## 5. Conclusions

The CREATE-based educational program was associated with significant improvements in evidence-based practice competencies among both nursing students and registered nurses in Sudan. Despite the challenges posed by a resource-constrained and fragile context, the program demonstrated the feasibility of delivering EBP education through a synchronous online format supported by local institutional collaboration. The integration of contextually relevant clinical activities, such as the development of a KMC protocol, may have enhanced participant engagement and facilitated the application of EBP principles. Although further research using more rigorous study designs is warranted, this experience provides a practical and potentially scalable model for strengthening EBP capacity in low- and middle-income countries, particularly in settings affected by humanitarian crises, conflict, or limited access to educational resources.

## Figures and Tables

**Table 1:** Educational Intervention Contents using the CREATE framework.

Dates	Programme	CREATE Framework Component	Main Content	Teaching and Learning Activities	Time	Teaching Team
Wednesday, 04 March 2026	Initial assessment	Baseline assessment of EBP attitudes, knowledge, skills, self-efficacy, and use	Initial assessment of participants’ EBP-related knowledge and competences	Administration of the pre-test questionnaires: - EBP-COQ Prof© - EBP-COQ	Before the lesson (about 30 min before)	- Cultural mediator - Two nurse research fellows (PhD, PhD student - Registered Nurse with expertise in pediatric, maternal, and child healthcare (MScN)
	Training module 1 part 1	Foundation for all CREATE steps	Foundations of research methodology	- Types of study designs and the evidence pyramid	2 h	
	Training module 1 Part 2	**Ask**	Searching for best evidence: framing the research question	Structuring the research question	2 h	
Thursday, 26 March 2026	Training module 2	**Search**	Searching for best evidence using MEDLINE via PubMed	Building a search strategy based on the structured question (from module no. 1) and running the search in MEDLINE via PubMed	4 h	
Thursday, 02 April 2026	Training module 3	**Appraise** **Evaluate**	How to collect quantitative data	How to collect quantitative data (examples related to: physiological changes in pregnancy; pregnancy nutrition—micro/macro nutrients; anaemia prevention; prevention of maternal complications: hypertension, infections, bleeding). Reliability of the data collection method	4 h	
Wednesday, 08 April 2026	Training module 4	**Appraise**	Quantitative data analysis	- Descriptive analysis - Correlations	4 h	
Wednesday, 15 April 2026	Training module 5	**Appraise Integrate** **Evaluate**	How to collect qualitative data	- Interviews - Focus groups - Field notes	4 h	
Wednesday, 22 April 2026	Training module 6	**Appraise** **Integrate**	Phenomenological qualitative data analysis; how to analyse qualitative data	Van Kaam Method as adapted by Moustakas and Content Analysis	4 h	
05–07 May 2026	Training Module 7	**Ask, Search, Appraise, Integrate, Evaluate**	Development of an internal protocol on kangaroo mother care	Participants formulated a research question, searched MEDLINE via PubMed, critically appraised retrieved papers, synthesized evidence, and developed a protocol on the use, benefits, indications, and precautions of kangaroo mother care	16 h	
After completion of the training	Final assessment	Post-intervention assessment of EBP attitudes, knowledge, skills, self-efficacy, and use	Final assessment of participants’ EBP-related knowledge and competences	Administration of the post-test questionnaires: - EBP-COQ Prof© - EBP-COQ	After the lesson (about 30 min after)	

**Table 2:** Socio-demographic and professional characteristics of nursing students and registered nurses participating in the EBP educational program.

Variable	Nursing Students (*n* = 12)	Registered Nurses (*n* = 17)
	M	±SD
Age (years)	24.2 ± 4.1	37.6 ± 10.2
Professional experience (years)	–	14.4 ± 10.0
	***N***	**%**
Gender		
Female	8 (66.7)	15 (88.2)
Male	4 (33.3)	2 (11.8)
Educational level		
Bachelor-level education	12 (100.0)	13 (76.5)
Postgraduate education (Master’s degree or PhD)	–	4 (23.5)
Place of residence		
Port Sudan	8 (66.7)	13 (76.5)
Other locations	4 (33.3)	4 (23.5)
Clinical training/practice characteristics		
Observed EBP implementation during clinical placement	8 (66.7)	–
Working in critical care settings	–	7 (41.2)
Working in maternal and child health services	–	8 (47.1)

**Table 3:** Pre–post comparison of outcome measures (*N* = 12).

Dimension	Pre Mean ± SD	Post Mean ± SD	Mean Difference (95% CI)	*t*(11)	*p*
Attitude	51.67 ± 4.38	55.25 ± 3.08	3.58 (0.85 to 6.31)	−2.89	0.015
Skills	17.33 ± 3.55	16.92 ± 2.57	−0.42 (−2.18 to 1.35)	0.52	0.614
Knowledge	15.33 ± 4.77	20.08 ± 2.02	4.75 (1.43 to 8.07)	−3.15	0.009
Overall Total Score	84.33 ± 7.73	92.25 ± 3.72	7.92 (2.79 to 13.05)	−3.40	0.006

**Table 4:** Pre–post comparison of EBP-COQ Prof© scores among registered nurses (*N* = 17).

Dimension	Pre Mean ± SD	Post Mean ± SD	Mean Difference (95% CI)	*t*(16)	*p*
Attitude	36.35 ± 2.89	37.53 ± 1.84	1.18 (−0.47 to 2.82)	−1.51	0.150
Knowledge	18.53 ± 5.44	29.88 ± 3.20	11.35 (8.68 to 14.02)	−9.01	<0.001
Skills	27.18 ± 5.73	35.76 ± 3.99	8.59 (5.61 to 11.56)	−6.12	<0.001
Utilization	42.18 ± 9.13	46.06 ± 8.45	3.88 (−3.26 to 11.02)	−1.15	0.266
Overall Total Score	124.24 ± 17.32	149.24 ± 13.78	25.00 (14.20 to 35.80)	−4.91	<0.001

## Data Availability

The data that support the findings of this study are available from the corresponding author upon reasonable request. Data are not publicly available because they contain information related to participants’ educational assessments and are subject to confidentiality and ethical restrictions.

## Abbreviations

The following abbreviations are used in this manuscript:

AICS	Italian Agency for Development Cooperation
AISPO	Associazione Italiana per la Solidarietà tra i Popoli (Italian Association for Solidarity among Peoples)
CET	Central European Time
CREATE	Classification Rubric for EBP Assessment Tools in Education
EBP	Evidence-Based Practice
EBP-COQ	Evidence-Based Practice Competence Questionnaire
EBP-COQ Prof©	Evidence-Based Practice Competency Questionnaire for Professionals
I-CVI	Item level—Content Validity Index
KMC	Kangaroo Mother Care
LMICs	Low–Middle Income Countries
MScN	Master of Science in Nursing
p.m.	Post meridian
PhD	Philosophy Doctor
SD	Standard Deviation
